# Supplementary material for: Cell-type diversity and regionalized gene expression in the planarian intestine
Source: eLife. 2020 Apr 2;9:e52613. doi: 10.7554/eLife.52613 (PMC7117911; doi:10.7554/eLife.52613)
Supplement: Transparent reporting form [file elife-52613-transrepform.docx]

***eLife’s* transparent reporting form**

We encourage authors to provide detailed information *within their submission* to facilitate the interpretation and replication of experiments. Authors can upload supporting documentation to indicate the use of appropriate reporting guidelines for health-related research (see [EQUATOR Network](http://www.equator-network.org/%20)), life science research (see the [BioSharing Information Resource](https://biosharing.org/)), or the [ARRIVE guidelines](http://www.plosbiology.org/article/info:doi/10.1371/journal.pbio.1000412) for reporting work involving animal research. Where applicable, authors should refer to any relevant reporting standards documents in this form.

If you have any questions, please consult our Journal Policies and/or contact us: [editorial@elifesciences.org](mailto:editorial@elifesciences.org).

**Sample-size estimation**

- You should state whether an appropriate sample size was computed when the study was being designed
- You should state the statistical method of sample size computation and any required assumptions
- If no explicit power analysis was used, you should describe how you decided what sample (replicate) size (number) to use

Please outline where this information can be found within the submission (e.g., sections or figure legends), or explain why this information doesn’t apply to your submission:

No explicit power analysis was used to determine sample sizes. For RNA-Seq, number of animals (4) and sections from each animal (10-20) used for laser capture is reported in figure legends and methods; RNA yields and quality for biological replicates are provided in Figure 1-figure supplement 1. All in situ hybridization experiments (except Figure 1-supplement 2) were conducted with 4-6 animals per riboprobe (and at least two replicates); this is also reported in methods. Sample sizes for analysis of *gli-1* and *RREB2* RNAi phenotypes are reported in legends for Figure 8 and Figure 8-figure supplement 6.

**Replicates**

- You should report how often each experiment was performed
- You should include a definition of biological versus technical replication
- The data obtained should be provided and sufficient information should be provided to indicate the number of independent biological and/or technical replicates
- If you encountered any outliers, you should describe how these were handled
- Criteria for exclusion/inclusion of data should be clearly stated
- High-throughput sequence data should be uploaded before submission, with a private link for reviewers provided (these are available from both GEO and ArrayExpress)

Please outline where this information can be found within the submission (e.g., sections or figure legends), or explain why this information doesn’t apply to your submission:

For RNA-Seq, we analyzed expression data from four (rather than three) individual animals, which we treated as biological replicates to increase statistical power when identifying differentially expressed transcripts; no samples were excluded from analysis. All in situ hybridization experiments (except Figure 1-supplement 2) were conducted with, and are representative of, at least two independent replicates with 4-6 animals per replicate/riboprobe; this is also reported in methods. Replicates for analysis of *gli-1* and *RREB2* RNAi phenotypes are reported in legends for Figure 8, Figure 8-figure supplement 6, and materials and methods, and source data are provided in Figure 8-source data 1. No outliers or replicates were excluded. Raw RNA-Seq data will be made available under NCBI GEO accession number GSE135351; reviewers may view this record using the private token below:

To review GEO accession GSE135351:
Go to [https://www.ncbi.nlm.nih.gov/geo/query/acc.cgi?acc=GSE135351](https://webmail.omrf.org/owa/redir.aspx?C=6KGFygNp7wSFICQRBgN-PF0eCXgE-XlOxs0k_e1l-OtVGuu9wV_XCA..&URL=https%3a%2f%2fprotect-us.mimecast.com%2fs%2fdfM7C9rG8Gikl1y5fEm5N7%3fdomain%3dncbi.nlm.nih.gov)
Enter token ctwpwumoppanbwb into the box

**Statistical reporting**

- Statistical analysis methods should be described and justified
- Raw data should be presented in figures whenever informative to do so (typically when N per group is less than 10)
- For each experiment, you should identify the statistical tests used, exact values of N, definitions of center, methods of multiple test correction, and dispersion and precision measures (e.g., mean, median, SD, SEM, confidence intervals; and, for the major substantive results, a measure of effect size (e.g., Pearson's r, Cohen's d)
- Report exact p-values wherever possible alongside the summary statistics and 95% confidence intervals. These should be reported for all key questions and not only when the p-value is less than 0.05.

Please outline where this information can be found within the submission (e.g., sections or figure legends), or explain why this information doesn’t apply to your submission:

Statistical analysis, significance testing methods, and dispersion/precision measures are reported in figure legends, source data, and materials and methods.

(For large datasets, or papers with a very large number of statistical tests, you may upload a single table file with tests, Ns, etc., with reference to sections in the manuscript.)

**Group allocation**

- Indicate how samples were allocated into experimental groups (in the case of clinical studies, please specify allocation to treatment method); if randomization was used, please also state if restricted randomization was applied
- Indicate if masking was used during group allocation, data collection and/or data analysis

Please outline where this information can be found within the submission (e.g., sections or figure legends), or explain why this information doesn’t apply to your submission:

For all experiments, all animals were randomly selected from large (300-500 animals) pools, and placed into experimental groups. No other allocation, randomization, or masking methods were used.

**Additional data files (“source data”)**

- We encourage you to upload relevant additional data files, such as numerical data that are represented as a graph in a figure, or as a summary table
- Where provided, these should be in the most useful format, and they can be uploaded as “Source data” files linked to a main figure or table
- Include model definition files including the full list of parameters used
- Include code used for data analysis (e.g., R, MatLab)
- Avoid stating that data files are “available upon request”

Please indicate the figures or tables for which source data files have been provided:

Figure 2–source data 1. Figure 6–source data 1. Figure 6–source data 2. Figure 7–source data 1. Figure 8–source data 1. In addition, raw RNA-Seq data will be made available under GEO accession number GSE135351.
